# Notes and Recollections of a Professional Life

**Published:** 1846-07

**Authors:** 


I. Notes and Recollections of a Professional Life. By the late *William Fergusson,* Esq. M.D. Inspector General of Military Hospitals. Edited by his Son, *James Fergusson.* 8vo. pp. 248. London, 1846. Longman and Co.

II. Inaugural Dissertation on Yellow Fever, and on the Treatment of that Disease by Saline Medicines. By *G. F. Bone,* M.D. Assistant Surgeon to the Forces. 8vo. pp. 85. Edinburgh, 1846. Black.

III. Correspondence on the subject of the "Eclair," and of the Epidemy which broke out in the said Vessel. Presented to the House of Commons by Command of Her Majesty, in pursuance of their Address of the 23rd January, 1846. Folio, pp. 94.

Dr. Fergusson was one of the most active and intelligent medical officers which our army has produced during the present century. He had seen a great deal of service in different parts of the world from the year 1794, when he joined the 90th regiment, as assistant-surgeon, at Ghent, in the unfortunate expedition under the Duke of York. Two years afterwards, we find him at St. Domingo, where he remained several years in active employment. On the final evacuation of that colony, he received thanks in public orders for his services. In 1801, he accompanied the expedition to the Baltic as Staff-surgeon of the troops embarked, and was on board the Admiral's ship on the occasion of the famous attack, under Nelson, upon Copenhagen. In the Peninsula, he was present at the taking of Oporto, and at the battles of Talavera and Busaco. In 1815 he accompanied the expedition against Guadaloupe, and then remained in the West Indies for two years. In 1817, he settled as physician at Edinburgh; but in 1821, finding the ground entirely pre-occupied in the northern metropolis, he moved his quarters to Windsor, where he eventually acquired an extensive and very lucrative practice, and where he continued to reside till the period of his death in January of the present year. Throughout the whole of his busy career, he had been an ardent and zealous writer upon medical matters; more especially upon those appertaining to the subject of Fevers, Contagion, Quarentine, &c. Many of his communications appeared in the pages of our respected cotemporary, the Edinburgh Medical and Surgical Journal. For some years before his death, he had formed the plan of collecting together his scattered writings, extending and enlarging them, and adding others, with the view of publishing them all in one substantive volume. This he was not spared to complete. The first half only of the present work was revised and corrected by the author; the other half consists entirely of re-published papers. The former is chiefly occupied with articles on subjects strictly military; as Military Tactics; the Exercises, Dress, and Arms of the Soldier; Barracks and Barrack-life; Military Hospitals and Staff; and Diet and Rations; there are also short articles on Fever as an Army Disease, on Disinfectants, and on Dysentery, Ophthalmia, and Syphilis. The latter part of the volume contains the author's papers on Plague and Quarantine, on Yellow Fever, on Typhus Fever and Contagion generally, on Marsh Miasmata, on the employment of Black Troops in the West Indies, and, lastly, his letters on Cholera. As a matter of course, we must pass over the military papers without notice; especially as we have already devoted a good many pages, in the present number, to one of the subjects discussed—that of Military Punishments. We are glad to find that Dr. Fergusson had taken the same enlightened and benevolent views upon the question of Flogging, as Dr. Marshall has done. What we propose to do at present is to select the most prominent facts and reasonings adduced by our author upon the very important subject of Fevers, more especially in reference to their direct transmissibility from one person to another, and the sanatory measures that should be adopted to check their diffusion.

The paper on "Contagion of Typhus Fever and Contagion generally," opens thus:—

"The true essential contagions, which, under a gaseous or aerial form, act of themselves independent of, and unaided by, the circumstances of climate, atmosphere, locality, quantity, and accumulation,—do not amount to more than five or six, and may all be comprehended under that class, of which it is the distinguishing characteristic, to occur only once, generally speaking, during the lifetime of an individual; with the exception always of those infections that can only be communicated by inoculation, or the actual contact of matter. I am far, however, from pretending to say that contagion is limited to so confined a range; for the whole class of *pyrexiæ*, under every shape and form in which they can be presented to us, including even those of erysipelas and ophthalmia, can be made infectious diseases, through an undue accumulation of human exhalations, and defective medical police, constituting at these times, and under these circumstances, an undoubted well-marked atmospherical contagion of locality,—but of locality alone.

"From the first of this enumeration I have no hesitation to strike out typhus fever, and class it amongst the latter." P. 162.

Typhus fever may be truly called an endemic of the British Isles. It seems to spring up spontaneously with each revolving season, under the operation of "moist cold, when applied to the destitute under circumstances of moral and physical depression." Extremes of temperature appear to resist its development; for it is but little known in tropical regions, except under circumstances of most defective medical police—even the Plague stops at the tropic of Cancer;—and it is equally rare in very high latitudes. Dr. F. is of opinion that the practice among the Russian peasants of heating their close, and anything but cleanly, habitations so much by the constant use of a stove night and day, as well as of so frequently making use of vapour baths, has unquestionably a salutary effect in counteracting the development of typhus. Besides the endemial cause alluded to, this fever is apt to arise from the mere accumulation of the effluvia arising from the human body, whenever masses of people are crowded together in a narrow space, while cleanliness and due ventilation are at the same time neglected. Hence the frequent prevalence of the disease in prisons, ill-constructed hospitals, on board certain ships, &c.: and hence, too, the not uncommon circumstance of its becoming superadded to, and, as it were, engrafted upon other maladies—such as the various forms of remittent fever, dysentery or diarrhœa, influenza, &c.—if the patients be unfavourably situated or improperly treated. Dr. Fergusson expresses the sentiments of a very large proportion of his professional brethren, when he says: "Diseases, which in their origin are beyond all doubt non-contagious, may temporarily acquire that property from generating a contagious atmosphere amid multitudes of the sick, and high concentration of what was the original cause during the progress of an epidemic."

That the contamination of the air with human effluvia will give rise to a truly infectious miasm, is abundantly proved by the history of Hospital gangrene or Contagious ulcer.

"This ulcer is precisely a local form of typhus fever,—a visible incarnation, if I may use the term, of the typhoid poison. It never occurs but under the most distressful crowding of sick and wounded; and it is then so highly contagious, that all other ulcers, or even abrasions of the skin, however healthy before, are speedily involved in its destructive course: and so highly does it impregnate the surrounding atmosphere with its contagion, that it is not even safe to bleed a patient in the same ward where it lies. You may look in vain for its origin under any circumstances in our hospitals, but those just enumerated, as being capable of inducing typhus fever upon the sound healthy inmates; but in the wounded, where the poison finds a nidus and a vent, instead of affecting the constitution generally, it commits its hideous ravages upon the wounded limb.*" P. 164.

That Typhus fever is apt to become contagious, although it may not be so primarily or intrinsically, cannot be reasonably disputed by any candid and experienced observer. Nevertheless, while we admit the truth of this, it should never be forgotten that the disease will seldom or ever spread when the patients are removed from the original *habitat* of the evil and placed in airy and clean dwellings. In this respect, therefore, typhus is essentially distinct from small-pox, scarlatina, pertussis, and other intrinsically contagious diseases; which, it is well known, often prevail over the entire extent of a district and among all classes of its inhabitants, unopposed, even in the slightest degree, by any sanatory regulations that can be adopted. Not so with typhus. The risk of infection from it is, *cetæris paribus*, nearly exactly proportionate to the impurity of the atmosphere, and the absence of personal cleanliness; although we must also admit that the disease appears to be infinitely more malignant and communicable in some seasons than in others.

The mere circumstance of a fever spreading from one person to other residents in the same infected locality is certainly no proof of its contagion; for all are equally exposed to the same morbific influence. Unless the patient can carry the disease to a distance and there communicate it to

---

* "The above relates to hospital gangrene alone, and has no reference to endemic or constitutional ulcers, which, however formidable their ravages may be, are never in themselves contagious. It may, however, reconcile my readers to the above doctrine, to state the fact that ulcers, devastating ulcers, have been seen in many parts of the world to be the substitute for endemic fever. Our army in St. Domingo, during the years 1796-7-8, abounded with such proofs; and in some parts of the East Indies, where I have never been, I understand that examples of it are even more rife."

others dwelling in a purer air, we cannot fairly recognise the existence of decided contagion. " In this transportability resides the very touch-stone and answer to the question of contagion ; to talk of contagion limited to one spot is merely only saying that the spot of ground, and not the person of the patient must be the source of the disease."

It seems probable that, when typhus arises from the effluvia generated in, and proceeding from, the human body in circumstances unfavourable to cleanliness and free ventilation, rather than from any merely endemic or local cause, the disease is much more liable to acquire a contagious character. The following remarks on this point will be read with profit:—

" That other form of typhus fever, which I may call factitious, (as being created by ourselves out of causes over which we ought to have exerted due control,) to distinguish it from the endemic, which we cannot prevent, must also be contagious under the same circumstances. Here, however, I believe, in like manner, that the person of the patient, independent of *fomites*, never gives out at any one time a sufficiency of the typhoid poison to affect another healthy person; that the poison can only be made effective through contamination of atmosphere, under long-continued accumulation of morbific effluvia; and in fine that the atmosphere of the patient is infectious, and not his person, which, if once cleansed and purified, and ventilation restored, may be approached, however ill he may be, with perfect impunity. In this belief I feel warranted, from the knowledge of several important facts, of a character so general as to warrant the greatest confidence in their application. 1st, The Bristol Hospital, for a great many years, has received typhus fevers into its well-disciplined wards, without having ever spread the disease even to the most contiguous beds. 2nd, Several of the great hospitals in London have followed the same example with the same results. 3d, The most pestilentially dangerous to approach when single in the confined dwellings of the poor, have almost everywhere been found devoid of every infectious principle when collected together in numbers, and confined by the hundred within the walls of a well-regulated fever hospital. Upon this point the question of contagion must turn; for, if the evidence here given be not impugned, it will be impossible in human testimony to adduce anything more decisive and conclusive. Reasoning from single individual instances will generally deceive; but well-digested impartial observation upon masses of men can never lead to an erroneous conclusion.*" P. 172.

In reference to the admission of fever patients into general hospitals, we may remark that it has been found safer and better to have them scattered as single eases through different wards, instead of congregating them together into one. This plan has been adopted in more than one of the London Hospitals; and we believe that there has been, on the whole, no cause to regret having followed it.

Every one, who has written on the subject of contagion, has recognised the powerfully predisposing effects of whatever tends to depress the energies of the mind and body.

---

* " The author alludes here to what he witnessed more especially at Liverpool, when on the staff of the north-west district, in the year 1804. Typhus fever had, up to that time, proved a most virulent and dangerous contagion to the medical faculty visiting the poor in the lower part of the town, who lived for the most part in cellars or other wretched dwellings : but on the building of an excellent Fever Hospital its character there as a contagious disease altogether ceased."

"There is still another principle bearing resemblance to contagion, and affecting the health of masses or bodies of men, of which, as it acts upon the fluctuating bases of moral and mental agencies, it is difficult to give any intelligible account. The health of a ship's company, for instance, or a corps of troops, when the *morale* is good, the discipline strict but kind, and the confidence of the men assured, will often be found firm in the midst of endemic and epidemic diseases; and plague and pestilence seem to pass them harmless until the introduction of unseasoned recruits, even though healthy at the time,—change of system, or some other cause, makes a break into the general health; and then will the whole rush into disease with a proclivity of current fully as remarkable as the preceding immunity. It would be ridiculous to say that this can be similar to what we often witness in *Chorea Santi Viti*, or the imitative hysteria of girls, or the familiar acts of yawning, stuttering, &c. Yetit may be akin in a certain sense; since all our sympathies are contagious, as the word itself implies; and when the protecting shield has been removed, these seize the reins, and guide the man without restraint. So fully, indeed, do they possess him, that when his mind is impressed with the dread of impending pestilence, his ordinary diseases, it is well known, will be suspended, and, when it actually arrives, will be merged into the vortex of the new epidemic." P. 178.

There is much good sense and practical value in the following observations on the proper method of arresting the diffusion of typhus, and other contingently contagious diseases:

"Could we establish the point, that this febrile contagion, unlike the poisonous leaven of the variolous fluid, or the venomous fluid of the viper's tooth, which on the reception of a particle contaminates the whole mass, is an infection of accumulation and quantity alone, we shall have attained a degree of security of nearly equal importance to mankind as the discovery of inoculation, or the vaccine preventive itself; for then, instead of man constantly generating a poison which would eventually be fatal to his race, the typhoid virus, without any great stretch of fancy, may almost be taken for the monitor of his life, and preserver of his existence in its due integrity; because that accumulation and quantity, to the degree that can constitute a contagion, being incompatible with the wholesome decencies of life, ought to find no toleration in civilized communities, and may always be obviated by the simplest precautions of domestic police. When these are neglected, the powers of mind and body bestowed by the Creator languish, and are deteriorated, like unhealthy plants deprived of their nourishment in a deficient soil, or of the air and light necessary to their well being; for it has been wisely ordained, that man shall use his faculties to uphold and improve the station that has been assigned to him; and the behests of Providence can never be despised without incurring the appropriate penalty in the diseases that afflict him, and the various plagues that beset his life. It will be vain for him, and his rulers more especially, to plead contagion as a predicament from which they could not escape; for that very contagion is part of their fault, being nine times in ten the work of their own creation, through default of humanity, and in consequence of their crimes. Wherever good government prevails, the worst contagion that ever appalled an hospital may be dissipated by the simple separation of the inmates; and the most saturated lazar that ever came out of a pest-house may be disinfected in the burning of a basket of charcoal, putting to flight at once by this simple process the ill-omened hosts of quarantine throughout every nation, with all their vexatious machinery of imprisonment, fumigation, and delay." P. 181.

We pass on to consider what Dr. Fergusson says respecting

### The Yellow Fever.

It rarely affects the Creolised white inhabitants of the West Indies, and the coloured classes never. How is this?—asks our author. Import among them small-pox, or any truly and essentially contagious disease, and they will suffer even more than Europeans. Take them to England, and they are as liable, as ourselves, to be affected with typhus. The truth is, that yellow fever " is a seasoning fever of malignant type, the product of high temperature, and unwholesome locality alone."* As a matter of course, the residents are not so much affected by those influences as new-comers from a temperate climate. To show how little contagion has to do with the diffusion of yellow fever, Dr. F. asserts, as the result of his own experience, that the medical men are not usually more liable to its attacks than the other officers of a regiment, and that the orderlies and immediate attendants upon the sick in hospital—provided always this be situated in a healthy locality and be kept cool, clean, and well ventilated—invariably suffer less than the soldiers in barracks. The reason is pretty obvious; the former are not so much exposed to the sun during the day and the dews that fall at night, nor yet to the chances of drunkenness and other mischievous excesses. Again, a vessel, while at anchor in one of the West India ports, may be daily losing some of her crew from yellow fever. No sooner does she set sail, and get a little to the Northward, than all trace of the disease usually ceases. As for the alleged transportation of the pestilence from the West Indies to Gibraltar, Cadiz, or any other European port (where it has been known to prevail), the idea is, in our author's opinion, utterly untenable. These places may produce, or be visited by, the very same malarious influences which gave rise to the fever in the New World. As the typhus of our own country cannot be transported to the West Indies, so the endemic of the latter cannot be transported to, or at least established among, us. A tropical climate is necessary to its existence. These remarks of our author, we need scarcely say, must be received with a good deal of qualification. We shall see immediately how far the history of the "Eclair" fever tallies with some of them.

Is Yellow Fever merely an aggravated form of the Remittent Fever of most tropical countries? or is it primarily and essentially a different disease? Dr. F. frankly admits that he is unable to decide the question. There are several circumstances connected with the history of the former—more especially the fact of its unexpected outbreaks in particular seasons which exhibit no appreciable peculiarities, and its equally inexplicable subsequent lulls, while the invasions and departure of the latter are annual and can be calculated with something like certainty; not to mention also the singular circumstance of the black-vomit fever being confined to the African coast and the West Indies, and scarcely if at all known in the East—which must make the cautious enquirer pause before he takes upon himself to pronounce a positive opinion. In considering this question, it should not be forgotten that we are often very much puzzled to account for the exceeding malignancy of certain epidemics of scarlatina, measles, puerperal fever, erysipelas and so forth; while, in other seasons, these diseases, though still

* This definition may fairly be objected to; but we are to remember that the present work is a posthumous publication, and we must not therefore look for the same accuracy and precision of expression that would have been expected, had it been otherwise. Dr. F. himself subsequently disclaims the opinion that yellow fever is merely a *seasoning* fever.

existing, are comparatively mild and innocuous. To add to the difficulty of solving the problem, Dr. Fergusson states that the Yellow and the Remittent fever often prevail at the same time, running side-by-side of each other. But, whether we regard them to be identical or not, "one point has been clearly ascertained, that there is no contagion whatever appertaining either to the one or the other." The following passage is valuable, from the interesting illustration that is adduced.

"No experienced men, unblinded by the prejudices of the schools and authorities, or biassed by the expectation of quarantine office, can seriously believe it (yellow fever) to be a contagion. It is a terrestrial poison which high atmospheric heat generates amongst the newly arrived, and without that heat it cannot exist; but it affects no one from proximity to the diseased, and cannot be conveyed to any low temperature. This was finely exemplified at Port au Prince, St. Domingo, where I spent the earlier months of the year 1796. Our head-quarters were the town and its adjunct, Brizzoton, as pestiferous as any in the world, and there we had constant yellow fever in all its fury. At the distance of a mile or two, on the ascent up the country, stood our first post of Torgean, where the yellow fever appeared to break off into a milder type of remittent. Higher up was the post of Grenier, where concentrated remittent was rare, and milder intermittent, with dysentery, the prevalent form of disease; and higher still was Fourmier, where remittent was unknown, intermittent uncommon, but phagedenic ulcers so frequent as to constitute a most formidable type of disease; and higher still were the mountains above L'Arkahaye, of greater elevation than any of them, far off, but within sight, low down in what was called the bight of Leogane, where a British detachment had always enjoyed absolute European health, only it might be called better, because the climate was more equable than in the higher latitudes. Here were the separate regions or zones of intertropical health mapped out to our view as distinctly as if it had been done by the draughtsman. Taking Port au Prince for the point of departure, the three first could be traversed in the course of a morning's ride. We could pass from the one to the other, and, with a thermometer, might have accurately noted the locale of disease, according to the descending scale, without asking a question amongst the troops who held the posts: and what kind of contagion must that be, which, amongst men in necessary intercommunication, cannot be conveyed from the one to the other, which refuses to mingle with another of lower temperature, although within sight, and so near, topographically speaking, as almost to touch? The men could, and did, constantly exchange duties, but not diseases; and it was just as impossible, and more so, to carry a yellow fever up the hill to the post in sight, as it would have been to escape had they been brought down and located amidst the swamps of Port au Prince. These things were known to every person in the army, whether medical, civilian, or military, and amongst them all there was not to be found a single person who had the smallest belief in contagion, provided always he had been a year in the country, and possessed opportunity of seeing with his own eyes,—all, I may say, came out contagionists, myself amongst the number, none remained so." P. 153.

Dr. Fergusson adduces a variety of considerations to shew that Yellow Fever is never essentially and intrinsically contagious. That it may acquire contagious properties, when the air, in which it was first developed, becomes vitiated with emanations from the sick, is highly probable; but this is an adventitious, not a necessary, property of the disease. The very exemption of the negroes from its morbific influence is a strong argument in favour of its being not contagious at first. Then, again, it is limited, in a great measure, to particular localities, temperatures, and elevations—

a character not very consistent with essential contagiousness. "Places, not persons," constitute the rule of its existence. It prevails during the highest tropical heat—the very condition that usually arrests the progress of infectious fevers, such as the plague (which usually ceases on the advent of Midsummer) and typhus.

In reference to the proper means to be taken to mitigate and arrest the pestilence, Dr. F. most justly remarks :—

"To pen up the inhabitants upon the infected ground is to aggravate the disease a thousand-fold, and is, in fact, as cruel and absurd as it would be to barricade the doors against the escape of the inmates of a house that had taken fire, on the insane pretence that they would otherwise spread the conflagration. The quarantine authorities will no doubt interpose to save the world from the dire contagion : but let them be referred to the annual epidemic at New Orleans for information how often the yellow fever has been conveyed from thence to the upper settlements on the same river, to which the fugitives, sick and well, uniformly fly for refuge, or even to the steamers that carried them; how often at Vera Cruz it has been carried out of the town even to the first stage on the road into the interior; or how often in Spain it has ever been transported, except to another station under the same circumstances, of heat and drought and defective perflation, therefore falling, if not having already fallen, into the same predicament from the same causes. Let them inquire whether seclusion and shutting up has ever saved the terror-struck. In the army of St. Domingo it was notorious that they were ever the first to be taken ill, and the surest to die; and during the yellow fever epidemic of 1816, at Barbadoes, I have recorded remarkable instances of the same both from my own observation and that of others. In short, whenever the endemic agency could not be avoided, the best place of safety was often to be found in the sick apartment, for there quietude reigned, and fatigue, exposure, and intemperance, were most likely to be avoided." P. 158.

Drs. Bone, father and son, are as decided anti-contagionists as Dr. Fergusson; and, as the former has had a long and most extensive acquaintance with the diseases of the West Indies, his testimony must carry very considerable weight. We may quote the following passage from the inaugural dissertation of his son upon this point :—

"In hospitals that are well constructed, and where a correct hospital discipline is enforced, the attendants of yellow fever patients are not liable to the disease.

"The Naval Hospital, Barbados, was built by Admiral Cochrane, and was well constructed; each orderly employed in the wards had a sleeping-room partitioned off from the ward. The sick of the Pyramus frigate and of detachments were treated in that hospital in 1821 and 1822. The total number of persons treated in the hospital from 21st of June, 1821, to 22d February, 1822, was 243. Of these, 101 were fever patients, and 38 were convalescents from fever. None of the patients under treatment for other diseases caught yellow fever; none of the servants, 38 in number, or of the other persons employed in the hospital, 53 in number, were affected with yellow fever. Twenty-two persons died of yellow fever, and were all, except one or two, carefully examined after death by Dr. Bone or by his assistants, Dr. Bain and Mr. Campbell, and none of them caught yellow fever.

"But is there any danger in entering an hospital where there are patients with yellow fever? Yes, if that hospital be filthy, crowded, ill-ventilated, and the patients lying on the bedding they have soiled : for the air must be vitiated, deprived of its oxygen, charged with azote, and other pernicious gases, and therefore unfit for respiration; and the sight is shocking and the groans of the dying frightful; but were the patients affected with any other disease, or, on admission with no disease, and similarly circumstanced, the danger from breathing the vitiated air among them would be equal." P. 21.

Both Dr. Bone and Dr. Fergusson expressly assert that the disease is apt to be generated on board-ship, in tropical climates, from an impure state of the hold. The latter, alluding to the cases of the Regalia transport from the coast of Africa, and of the Childers sloop of war—of which he has given an account in the 8th volume of the Medico-Chirurgical Transactions—assures us that, whenever the holds of these vessels were cleaned and purified, the fever ceased. " Before that was done, whatever stranger slept in either of them for a single night, was assuredly taken ill; whoever was brought sick ashore into the fully occupied wards of our general hospital, and received with open arms, brought with him as little infection as the new-born babe."*

We may point to the testimony of Mr. Birtwhistle also, late surgeon of H.M.S. " Volage," as given in a recent number of the Lancet (3rd Jan. 1846). In the Spring of 1842, yellow fever having broken out in this vessel at Port Royal, she was ordered by the commodore on the station to proceed at once to Halifax. For some time after her departure from Jamaica, the disease prevailed, and indeed with increased violence, on board; for fresh cases continued to be added to the list even for some time after the ship's arrival at Halifax, and did not completely cease until all hands were landed on Navy Island. Let us now see what opinion Mr. B. formed as to the producing causes of the fever, and consequently as to the best means of arresting its diffusion.

" After maturely reflecting on the origin, progress, and termination of this dreadful malady in the 'Volage,' and weighing well everything which could in any way tend to produce or aggravate it, I must confess that it is difficult to come to a perfectly satisfactory conclusion; but it is, I think, evident that the primary cause was our lengthened stay at Chagres, Carthagena, and Santa Martha, where the fever first seriously presented itself, and where all the sources of malaria are most abundant; and that the manner in which it subsequently raged amongst the crew, first attacking the officers and servants residing in the steerage and after-part of the vessel, and thence proceeding to the adjoining mess of the marines, only one of whom escaped, and afterwards, forwards to the seamen, clearly indicate that in the vicinity of the pump-well there was some fatal miasma arising, which could not be, and was not removed until the thorough cleaning, drying, whitewashing, and fumigating, which she received at Halifax, all the stores and tanks being taken out for the purpose, and the holds being kept empty for nearly a fortnight. I had the gratification to find that the removal to the shore, and cutting off, for a time, all communication with the vessel, proved a most effectual remedy, inasmuch as from that period I had not a single new case, and the sick regained their strength in a rapid and surprising manner."

He adds :—

---

* We need scarcely say that Dr. Fergusson rejects the idea of Cholera being a contagious disease : " always however with the proviso and exception of the possibility of its being made a temporary contingent contagion amidst filth and poverty, and impurity of atmosphere from overcrowding and accumulation of sick, but neither transmissible nor transportable out of its own locality through human intercourse."

"It will be perceived that the fever had gone on unchecked for nearly three months, the latter cases, at Halifax, being as urgent and dangerous as those which took place at the beginning, and from its great severity, and almost unexampled duration, conjoined with the facts previously mentioned, it is, I think, demonstrated that the cause existed in the vessel herself; and this opinion is further confirmed by the circumstance, that two officers came on board to assist us, both of whom were taken ill within four days, and the same occurred to two others who were only on board an hour or two on a visit to some friends, having been exposed, of course, to the same cause which operated on our own men. No one, therefore, could join the ship with impunity, although I am satisfied that the whole of the sick might have been landed in any part without the slightest risk to the inhabitants.

"As to the question which has been so much discussed, and upon which such discrepancy of opinion exists—I allude to whether this affection can be imparted from one person to another,—it will be observed, that I am a non-contagionist, but I am, at the same time, not prepared entirely to deny its communicability, especially situated as we were in the 'Volage,' with so many cases of a malignant character crowded into such a small space, and where the air, in spite of every precaution, was impure and offensive." P. 8.

After the details which have now been given, touching the very important question of the contagiousness of the yellow or black-vomit fever, our readers will be the better prepared to follow and appreciate the melancholy history of the "Eclair epidemy," which we proceed to lay before them.

The "Eclair," a new steam-sloop, left England under the command of Captain Estcourt for the coast of Africa, in Nov. 1844, with a crew of 146 officers and men. She reached Ascension on the 1st of December, and Fernando Po by the 25th. During the next two months, she kept cruising along the coast, anchoring every now and then for a short time at different places. On the 23rd of February she was at Sierra Leone, and took on board the extra complement (40) of Kroomen and liberated Africans allowed on the coast. She remained there a few days, and then returned to Seabar, off the island of Sherboro. At this time, the men were much employed in the boats, blockading the place, and watching the movements of the slavers. They were often away for several days at a time, and had to sleep either in the boats or on shore, thus exposed to a tainted nocturnal atmosphere after a fatiguing day's work at the oars. It would seem that the men were sent upon this service six different times, and were absent altogether 29 nights. On some of these occasions, they were exposed to the heavy rains that fall at that season of the year. It deserves notice, at the same time, that in the place where the Eclair was anchored, the water was excessively filthy, from the washings of the mangrove marshes. The fresh water, used on board, was also very bad in quality. Several men suffered from diarrhœa and mild attacks of cholera. Besides all this, we learn that a degree of mental despondency prevailed among the crew, increased by seeing prizes captured by other vessels of the squadron frequently pass within sight, on their way to Sierra Leone.

Notwithstanding these many injurious influences, no case of decided fever occurred for five or six weeks at least. The first case occurred on the 3rd of April; it was severe, but the man recovered. On the 18th, three men, who had been much away in the boats, were seized: one died on the 5th, and two on the 6th day of the attack. The type of the fever was well-marked Remittent, often exhibiting very distinct intermissions. The head was generally much affected. No mention indeed is made, in the medical report of the ship, that the matters vomited were of a black colour; but it deserves especial notice that, in the account of three *post-mortem* examinations at this period, the following entries were made by Dr. Maconchy the surgeon—"the stomach contained some reddish glairy matter"—"the stomach was distended with dark serous fluid mixed with black flakes"—"not a clot was seen in the body, but fluid blood exuded from the nostrils and blistered back." The average duration of the fatal cases seems to have been five or six days.

From the last date up to the 22nd May, nothing particular seems to have occurred. The steamer continued to move about the coast; only one case of fever occurred, and the patient did well; and, although many of the men suffered occasionally from slight diarrhœa, bilious vomiting, and such-like ailments, there was no serious illness on board. On the 22nd of May, three new cases of fever were entered on the sick-list: they all proved fatal: one on the 5th, another on the 7th, and the third on the 13th day. Between this date and the 8th of June, other five cases occurred: four of the patients died in periods varying from three to nine days. Most, if not all, of the men seized had been engaged in the boat-service. The fever after this seems to have ceased for several weeks, until the Eclair again reached Sierra Leone. While there, part of her crew were employed in cleaning out the hold of the "Albert" steamer, which was in a most offensive state; it was supposed that nothing had been done to it since the vessel had returned, two years before, from the ill-fated Niger expedition. Moreover, the men sent on board the Albert seem to have had the opportunity of indulging in the most shameful intoxication; and, to add to their danger, permission was most unfortunately granted to them to go on shore, where some of them stayed all night, exposed to the most pernicious influences. On the 19th (of July), the fever re-appeared on board the Eclair: the patient died on the eighth day of the attack. During the next three days, three cases more occurred; and all proved fatal. On the 23rd the ship proceeded to sea, having the Albert in tow, and with twenty of her crew on board that vessel. They proceeded together to the northward, and remained at anchor off the coast from the 28th to the 8th of August; during which time a working party from the Eclair, in addition to the twenty already noticed, were employed on board the Albert. After leaving Sierra Leone, three fatal cases of fever occurred in July. A merchant, who had embarked on board the Albert at Sierra Leone, died on board that vessel on the 27th.* On the 1st of

* In the official report by Sir William Pym and Mr. (not Dr. as it is printed) Arnott, to which we shall afterwards have occasion to refer, it is expressly stated that "four days after sailing from Sierra Leone, one man died with fever and black vomit, the first case of the kind which had taken place: this man had been brought on board on the morning of the 23rd, having been the three previous days on shore." No specific allusion is made to this case in the subsequent report of Dr. Stewart, which professes to give an account of the health of the Eclair from the 26th of August 1844, to the 15th of Nov. 1845; nor indeed is any mention made in it when the first case of decided black vomit occurred. All that

August, other two cases occurred; but they did well. On the 2nd, there were two fresh cases: they proved fatal. On the 8th and 9th, four men fell sick; two died, and two recovered. (We have not been able to make out whether all these cases occurred in the Eclair, or whether some were on board the Albert.)

On the 10th of August—a most unhealthy time of the year on the African coast—the vessels arrived in the Gambia; the Albert was given up to the government there; and the Eclair remained until the 14th, when she sailed for Goree, having four sick of the fever on board. On arriving at Goree, she was refused pratique: one man died there. On the 17th, she proceeded to Bona Vista, one of the Cape de Verde islands, which she reached on the 20th: three men had died on the passage. Pratique was at once offered by the Portuguese authorities; but Captain Estcourt very frankly acknowledged that he had been put into quarantine at Goree in consequence of the fever on board, and he accordingly declined the generous offer of free admission until a sufficient enquiry had been instituted. Dr. Kenny, a resident English medical man, was commissioned by the governor to report upon the state of the Eclair. Upon consulting with her medical officers, this gentlemen said that the disease on board was the common coast fever of Africa, and that there was no risk in granting pratique. This was accordingly done. As the fever continued to prevail —for five fatal cases occurred during the ten days after the arrival at Bona Vista—Captain Estcourt was anxious to have his own men landed, and the vessel completely overhauled. The Portuguese official physician as well as Dr. Kenny, being consulted by the governor as to the safety of granting the permission desired, gave their complete sanction to the proposal; the former gentleman emphatically saying: "no danger at all; I have often brought sick men ashore coming in vessels from the African Coast, and I never knew any ill-effects to arise." Accordingly, not only was Captain Estcourt's wish granted, but a small fort, situated on an island at the entrance of the harbour, was also most generously given up for the use of the sick; while the officers, &c. were permitted to reside in private dwellings in the town. Scarcely a single man was in perfect health at this time; all were ailing more or less, so that there were not hands enough to do the work of the vessel. The surgeon, Dr. Maconchy, was worn out with fatigue and anxiety; and the same was the case with Captain Estcourt, whose conduct towards his sick men appears to have been truly noble. The Eclair remained at Bona Vista from the 21st August to the 12th of September. While there, the hold was thoroughly cleared out, the tanks removed, &c., by the Kroomen on board, the crew having already been landed. The hold, we are told, was found to be clean, although very

---

is said by Dr. Stewart on the subject is—just before describing the arrival of the Eclair at Bona Vista—to the following effect: "The fever appears to have been as distinctly remittent as that at Sherboro, and some of the men had unequivocal black vomit." It seems, therefore, that we cannot with accuracy determine when the first case of decided black vomit occurred. Sir William Pym and Mr. Arnott must have derived their information from the acting Commander Harston and Mr. S. Barnard the Surgeon; but then we are to remember that the latter gentleman only joined the Eclair at Madeira.

badly ventilated.* A gang of Portuguese labourers, about 30 in number, was employed on board the ship, in moving, hoisting, unloading, &c.; but none of these men, we are informed, went into the hold. The soldiers of the fort, too, communicated freely with the British sailors domiciled there. Notwithstanding the removal of the sick on shore, the fever continued to prevail and indeed to increase among the men. No fewer than 28 deaths took place from the 31st of August (the day of landing) to the 12th of September; making in all 39 deaths from the time of leaving Sierra Leone. We shall afterwards see that it is stated by Sir William Burnett that the men committed the greatest excesses in drinking at Bona Vista.

While the Eclair was at Bona Vista, the "Growler" steam-sloop, Captain Buckle, arrived from Sierra Leone on the 6th, just as the assistant surgeon of the former, Mr. Hartman, was taken ill: he died subsequently. Dr. Maclure, a naval surgeon, passenger on board the Growler, generously offered his services to replace his professional brother; the offer was at once accepted. For the next five days, Dr. Maclure was in constant attendance upon the sick in the fort. As the purser of the Eclair had died, an inspection of her stores was made by order of Captain Buckle; the senior officer, a lieutenant, the paymaster, the purser, and the clerk of the Growler officiating.

On the 12th of September, all the sick and the rest of the crew having returned on board the Eclair,† both steamers sailed from Bona Vista for Madeira, where they arrived on the 20th, but were refused pratique. During the passage, several deaths took place. Captain Estcourt, who had sickened on the 11th, died on the 16th; Dr. Maclure on the 17th; and Dr. Maconchy on the 21st, while the vessel was in Funchal roads.

Mr. Sidney Barnard, who was returning to England in the "Rolla," having volunteered his services, was appointed surgeon of the Eclair *pro tempore*, and Mr. Coffey, assistant-surgeon of the Growler, was sent on board as assistant-surgeon.

On the 21st the steamers sailed for England. The Eclair anchored at the Motherbank on the 28th, with twenty-three of the crew on the sick list: five of these were in bed with the fever, and the others (convalescent) in a very weak state. No fewer than 41 fresh cases of the fever had oc-

* The Eclair was very badly constructed for ventilation. The main deck forward was found to be so close that Captain Estcourt had ordered two circular openings, each about 16 inches in diameter, to be made on each side of the forecastle for the purpose of admitting air more freely. The lower deck was dark and badly ventilated; and from the very awkward position of the hatchways, these being placed not immediately the one over the other, wind-sails could not be used with much affect to send air down. Moreover, there were no gratings in the floor of the main deck, nor any copper tubes, (as in some ships) leading from the lower to the upper deck. The fore and after holds were utterly out of the reach of free ventilation. "Indeed," Dr. Stewart remarks, "it seemed as if there had been a studied avoidance of having the hatchways of the different decks in the same lines."

† A council had been previously held by the surgeons of both vessels, at the desire of Captain Buckle, to determine upon what steps should be taken. It was unanimously agreed that the Eclair should proceed without delay to England.

curred from the time of leaving Bona Vista; and, of these 41, 12 had proved fatal.*

While at the Motherbank, two men were taken ill and died. On the 1st of October, a pilot having been taken on board, the Eclair was ordered round to the quarantine ground at Standgate Creek. Upon arriving there, all those of the crew, who had escaped the fever (41 in number), were removed on board one of the line-of-battle ships in ordinary, the "Revenge;" while such as had recovered from it, and those of the convalescent as were in a state to be removed, were transferred to another two-decker, the "Benbow;"—leaving none on board the Eclair but the sick, the Kroomen (not one of whom had suffered), and the acting Commander.

Between the 1st and the 9th of October seven fresh cases occurred; of these, three proved fatal. Mr. Barnard, the surgeon, was among the number; he sickened on the 4th and diod on the 9th. The pilot,† who was one of those on board the Revenge, died on the 10th; and Lieutenant Isaacson, who was taken ill on board the Benbow on the 7th, died on the 12th. As the assistant-surgeon, Mr. Coffey, had had a slight attack on the 5th, it was necessary to supply his place by Dr. Rogers, the assistant-surgeon of the Admiral's ship at Sheerness; and, on the following day, Dr. Stewart was appointed surgeon of the Eclair. The former experienced a slight attack of the fever on the 10th; he gradually recovered. The same was the case with Mr. Coffey. No fresh case occurred afterwards; and by the 15th, all the sick were declared to be convalescent. On the 20th and the 21st, indeed, two men, who had acted as attendants upon the sick, and had afterwards superintended the clearing out of the Eclair's hold by the Kroomen, were seized with feverishness and irritability of the stomach; but these symptoms quickly passed away. Some of the Kroomen, too, had slight febricular attacks; but these might be owing rather to atmospheric vicissitudes than to any malarious agency.

On the 31st of October the Eclair was released from quarantine, Lieut. Harston (who had succeeded to the command upon the death of Com. Estcourt) having remained on board all the time.

Before alluding to the opinions of the official medical authorities at home touching the nature of the Fever of the Eclair, and the proper steps to be pursued under such distressing circumstances, we have a few words to say respecting the Growler, which, we have seen, was in company with the Eclair since the 6th of Sept. at Bona Vista. We have said that, while there, four of the officers of the former went on board the latter to make an inspection of her stores. Every one of them was attacked with the fever; one, the purser, was actually taken ill while on board the Eclair. All of these cases, as far as we learn, terminated favourably. One report states that not another case occurred on board the Growler: but

---

* During the stay at Bona Vista, no fewer than 60 fresh cases occurred: of these, 31 eventually died.

† This poor fellow seems to have died of alarm rather than of the fever. He had none of the symptoms of the disease; but he had scarcely eaten anything since he went on board the Eclair at Portsmouth. He sunk with symptoms of cerebral oppression, accompanying a sort of low typhoid fever. His constitution was very unsound.

Sir William Pym asserts that there were five or six other cases.* However this may be, it would seem that none proved fatal. The disease must therefore have been of a much milder character than that in the Eclair. The Growler arrived at Portsmouth on the 29th of September. Subsequently she moved round to Woolwich to be paid off. While she lay there, two men, engaged in clearing out the hold (which, it is stated, gave out a very offensive smell), and who had been sleeping immediately over the scuttles of the forehold, were seized with fever on the 10th Oct.; the cases were severe, and the patients were sent to the Marine Hospital on shore. Both died " with all the characteristic marks of yellow fever :" so says Dr. Stewart. Sir W. Burnett states that " the matter vomited approached to the appearance of black vomit, but not entirely so." The latter gentleman goes on to say that " the fever was *most decidedly* not of an infectious nature; no farther case having taken place in the Growler herself, or in any of the attendants at the hospital."

There is a communication, dated the 20th October, from the store-keeper of Woolwich Dockyard, that a quantity of the slop-clothing, &c. returned from the Growler had a most offensive smell. The stores were immediately ordered to be sent down to the quarantine station at Standgate Creek, to undergo the necessary purification.

It may be well here to notice that " since the arrival of the Eclair and Growler," we quote from a letter of Sir Wm. Burnett of the 29th Oct., " H.M.S. Ardent has also arrived with her crew in a state of perfect health, and without a single vacancy in her complement. I have also further to state, that by a report from the surgeon of Her Majesty's ship Penelope, the squadron employed upon the coast had not been visited by any unusual sickness during the previous six months (the period included by the return), and that the number of deaths from all causes did not exceed that of the most healthy years; nor is there the slightest reason to presume that any disease of a contagious nature prevailed in any part of the station."

Let us now see what measures, quarantine and other, were advised and adopted respecting the Eclair when she arrived in England.

Dr. Salter, of the Motherbank Quarantine office, was the first to report upon the state of the vessel. In his certificate, dated Sept. 28th, he states " that as many as 60 have died of fever, and that as many as 7 are still suffering from that disease. Three of the cases have occurred within the last week, and the type of the fever is of the most severe form—that accompanied with black-vomiting. Under these circumstances I have recommended the superintendent not to release the vessel. I cannot but consider the disease as contagious." Next day, Dr. Richardson, Inspector of Haslar Hospital, visited, or rather went to, the Eclair, by the command of the Port Admiral. In his report, dated Sept. 29th, it is stated that

* In one account we read that " there had been occasional attacks of fever for many months; viz. in May, 12 cases; in June, 7; in July, 5; in August, when they had been at Ascension, and only returned to Sierra Leone on the 26th of the month, 3; in September, 12; and in October, 2." No particulars, however, are given of the type of the fever, the mortality, &c.

"65 of the crew have died of the bilious remittent fever, endemic on the coast of Africa, that 5 of them died since the Eclair left Madeira, and one last night."

"Notwithstanding," continues Dr. Richardson, "the extraordinary mortality that has swept off so large a proportion of the crew of the vessel, I entertain no fears of her being the means of introducing epidemic disease into this country; and were the sick placed in well-ventilated wards, with fresh bedding and the other means of cleanliness afforded by an hospital, I anticipate no further risk to the attendants than would occur in wards set apart for cases of typhus fever; the decision on this point must rest, I conclude, with the Board of Quarantine; and should they decide on giving the 'Eclair' pratique, I should recommend the sick being removed to a wing of Haslar Hospital to be appropriated exclusively for them; and, lest alarm should be excited by any of the remainder of the crew being taken ill, it might be advisable to keep them on board their own ship for eight or ten days."

On the morning of the 30th, Mr. Arnott, the distinguished surgeon of the Middlesex Hospital, was sent down by Government, along with Sir Wm. Pym the Superintendent-General of Quarantine, to determine what measures should be taken. Mr. A., who had visited the Eclair before Sir W. arrived, had most judiciously arranged with the Admiral at Portsmouth to separate at once the sick from the healthy, by removing all the latter on board a frigate in ordinary. This most sound advice was not, however, carried into effect; as Sir Wm. Pym, taking into consideration the very sickly state of the crew, and the difficulty of communicating with the vessel at the Motherbank in boisterous weather, thought it better to order her round to Standgate Creek. What was done there with the crew, we have already seen. The report of Sir W. Pym and Mr. Arnott is dated Oct. 3rd.

On Oct. 7th, Sir Wm. Burnett, the Director-General of the Medical Department of the Navy, addressed to the Admiralty a letter, wherein he expresses a confident opinion that "the fatal fever on board the Eclair had been originally occasioned by the effects of malaria and exposure in the rivers, by the long continuance at Sierra Leone, and the many irregularities committed by the ship's company in that port." Sir Wm. continues: "It is necessary that I should add, that a fever, not originally of a contagious property, may become so when the sick are crowded together in a small, ill-ventilated place."

On the 22nd of October, Sir Wm. Pym wrote to Government on the subject of the late fever. The opening paragraph of his letter runs thus: "In consequence of the arrival lately of men-of-war steamers from the coast of Africa, whose crews had suffered from the disease known as the Yellow, Bulam, or Black-vomit fever, a disease of a highly infectious nature, much more to be dreaded than the plague, and, if imported into this country during the summer months, would occasion a most frightful mortality, and would for a long period of time prove disastrous to commerce, in consequence of the rigid quarantine which would be established in all parts of Europe, but more particularly in the Mediterranean, upon all vessels arriving from the United Kingdom. It is a disease of a warm climate hitherto unknown in England, and imported by means of an artificial warm climate having been kept up during the voyage by the fires of the steamers." To guard against the risk of importation, Sir W. Pym went so far as to propose that Government should give instructions to detain in future all men-of-war, and especially steamers, on the African and West Indian stations until the 1st of Nov.; so that they might not arrive in England before the winter months, at a season, "when the degree of cold would be such as is considered sufficient to destroy the infectious nature of the disease."

When this proposal was submitted to Sir W. Burnett for his opinion, it met with his strongest and most decided opposition; in which, it is but right to state, the Lords of the Admiralty expressed their entire concurrence. We shall make one or two extracts from Sir W. B.'s letter on the subject. After deprecating the proposal to detain men-of-war until November on the coasts of Africa and the West Indies, as fraught with great danger to the crews in consequence of the sudden transition from a tropical to a cold climate, he says:—

"It is perfectly evident from the history of the Eclair, and her proceedings on the coast, that the fever in question arose from causes totally distinct from infection, and that it was in fact the usual remittent fever of the coast, produced originally by the influence of marsh miasmata, heightened by the exposure of the men in boats, when absent from the ship for many days together, to miasmatic influence, and to the subsequent irregularities which the men committed in Sierra Leone, when they unfortunately obtained leave to go on shore, and where great excesses were committed—which combination of causes has never yet failed to produce a fearful increase of febrile disease, particularly on the coast of Africa. I may here add, that the increased mortality, which took place whilst at Bona Vista, is no longer a mystery; it was caused, I regret to say, by the most intemperate use of spirits I ever heard of. My informant told me that a bucketful of spirits had been offered to him. I do not mean to deny the possibility of this or any other fever becoming infectious under such circumstances as attended that in the Eclair; but there is not the least proof that it was so, while there are circumstances and proofs that inevitably lead to a contrary conclusion. But be this as it may, I have no hesitation in declaring my firm belief that the sick men of the Eclair, when that ship arrived at the Motherbank, might have been landed at Haslar Hospital, and placed in the well-ventilated wards of the establishment, without the public health suffering in the smallest degree."*

This opinion had been previously expressed, as we have seen, by Dr. Richardson.

Sir W. Burnett goes on to say: "It is a fact well known that, during the autumn of every year, merchant ships arrive in our harbours loaded with the produce of the coast of Africa, having perhaps lost great part, nay in some instances the whole, of their crew by the fever of the country; or some are still labouring under fever when the ship arrives in the Thames, and are sent to the hospital in that state: yet no instance is known of any infection having been produced by such procedure; in fact, it is perfectly certain that it never did take place."

It was but fair that Sir W. Pym should have an opportunity of replying

---

* At the same time, Sir Wm. suggested that his patent solution of Chloride of Zinc, which so instantaneously destroys foul smells, should be introduced into the hold, and applied freely to other parts of the vessel.

to the assertions and arguments of his opponent. In his reply, he contends that there are two distinct forms or types of fever prevalent on the coast of Africa; viz. the common Remittent, and the true and proper Yellow or Bulam fever.

The *former* is the same as the Walcheren fever, the malaria fever of the Levant, the jungle fever in India, &c. One attack does not protect from a second; and those, who recover from it, suffer almost invariably afterwards from ague. It is *not infectious.*

The latter is a disease *sui generis,* is in no way connected with malaria or unhealthy situations, is unknown in the East Indies, is peculiar to the West Coast of Africa, is *highly infectious,* and therefore has often been imported into the West Indies, Spain, the island of Ascension, &c. Its peculiar symptoms in fatal cases is the vomiting of a black coffee-ground looking matter: "this symptom does not exist in the remitting fever." Those who have recovered from it (the former) are protected from a second attack;* nor do they ever suffer from ague during their convalescence.

We need scarcely say that Sir W. Pym regarded the "Eclair" fever as belonging to this second class; and by a variety of arguments he contends that it had been kept up and diffused on board by direct contagion. The circumstance of the disease raging with increased violence after the men had been landed at Bona Vista is fairly urged as a strong proof of this opinion.

Sir W. Burnett, in a subsequent letter, stoutly, but in a somewhat too dogmatic, tone, utterly denies the accuracy of these distinctions attempted to be established between the (alleged) two kinds of fever; contending that "the more ardent form of yellow fever is a mere modification of the bilious remittent, so extensively known over all tropical regions, and hence can have no infectious properties, unless under such circumstances as an accumulation of sick in a crowded and ill-ventilated space'" He proceeds to remark: "With respect to the subject of the importation of the disease into various places, except in one instance, and that even is surrounded with doubts—I mean that of H. Majesty's sloop Bann†—I entirely disbelieve it.........I have caused the medical reports of the Jamaica Hospital for more than 20 years to be examined; and, though hundreds of patients with yellow fever in all its most appalling forms, including black-

* The truth of this is expressly denied by Dr. Bone: "The contagionist doctrine that yellow fever, of which black vomit and yellowness are diagnostic symptoms, affects a person once in life only, as a general rule, is true; for very few persons recover who have vomited black vomit, and of these few, it may not be possible to discover any who have subsequently vomited black vomit; but, if it is argued that a person, who has been affected with fever in one epidemic of yellow fever, is not susceptible of fever in another epidemic of yellow fever, or in he same epidemic, the argument is altogether wrong."

† There were many circumstances connected with the fatal epidemy which broke out in that vessel that bear a very marked resemblance to those of the fever on board the Eclair. That the Bann, which had suffered dreadfully during the voyage from Sierra Leone to Ascension, introduced the fever into that island, cannot well be disputed by any one who peruses with candour the official Report that was published at the time (1824) by Sir Wm. (then Dr.) Burnett. An exvomit, &c., have been treated in that establishment, not one of the medical officers in charge have even hinted at the disease being contagious."

It is certainly the opinion of a large majority of naval and military medical men, that the Yellow Fever of the West Indies is not primarily or essentially contagious ; and it is a circumstance sufficiently remarkable in reference to the "Eclair" fever, that not one of the, alas! too many medical officers on board of her seem to have held the opinion that the disease had spread by infection from one person to another. In the official report, that was drawn up and signed by the surgeon of the Growler, and by Drs. Maconchy and Maclure, at Bona Vista on the 12th Sept., wherein they recommend the immediate departure of the Eclair for England, not a hint is thrown out about the contagiousness of the fever. The extracts, which we have given from Sir W. Burnett's letters, abundantly shew the drift of his opinions upon the subject. While peremptorily denying the primary contagiousness of Yellow fever, he nevertheless admits that it may acquire this property under certain circumstances. When he wrote, nothing was known about the health state of Bona Vista after the departure of the Eclair; and he very properly attached great importance to what might take place in that island, as bearing upon the question of the contagion or non-contagion of the fever on board the vessel. The following passage occurs in one of his letters :

"If it can be fully and satisfactorily shown that any person, who had so visited the ship or tents where the sick were placed, contracted the fever in question and communicated it to others, and they to other persons in succession who had never visited the ships or sick, then there can be no reason to doubt the infectious nature of the disease; but if nothing of this kind has taken place, then the conclusion must be that the disease is not infectious, and is therefore incapable of being communicated; in either case settling this long-contested question."

Dr. Stewart also candidly admits (the news of the outbreak of the fever at Bona Vista had just reached England when he was finishing his report) that, "if it shall appear that the fever, with which the inhabitants of Bona Vista are afflicted, first made its appearance after a reasonable interval in any of those who had been in communication with the crew of the Eclair, either in the town or at the fort; or if it first appeared among any of the Portuguese labourers who were employed in the Eclair, and subsequently extended to the families of those labourers, or any of their acquaintance who visited them when ill," he would readily recognize the contagious character of the disease. He had previously leaned, we may remark, to the opposite opinion.

On the 22nd of December, Mr. Rendall, the British Consul at Bona Vista, wrote to Lord Aberdeen a letter, wherein, after detailing the circumstances connected with the arrival, stay and departure of the Eclair,

---

cellent analysis—written by the late Dr. Johnson—of this report will be found in the Medico-Chirurgical Review, for January 1825. Dr. Johnson wisely belonged to the moderate party, avoiding the excesses alike of the ultra-contagionists and the anti-contagionists, and advocating the doctrine of contingent or adventitious contagion. Many of his observations in the article alluded to will amply repay an attentive perusal.

he communicates the following particulars. The Eclair and the Growler left on the 12th Sept. Nothing occurred till the 20th ; when it is stated that one of the Portuguese soldiers, who had mixed with the Eclair's crew, had died in the fort which had been occupied by them. As some of the other soldiers fell sick, the fort was abandoned, and the sick men were brought to the town and lodged in a house near the beach. The weather at this time was extraordinarily hot, and much rain had fallen. It deserves also to be mentioned that a large quantity of stagnant water had collected at the back of the town, and that the streets were in a most filthy and offensive state. The fever began to spread daily more and more ; all the cases occurring within the immediate neighbourhood of the house where the first death had taken place. The native medical men still persisted in regarding the disease as not infectious. This opinion they continued to hold up to the 20th of November, when they openly declared that the fever was of a most malignant type and very contagious. "Up to the first week in December," writes Mr. Rendall, "the fever continued to rage; and, at that period, it had found its way into almost all the country villages —the deaths averaging seven or eight daily. The last report that I have received is to the 21st. instant (Dec.), to which date 250 had died." The chief mortality was among the lower orders, whose state was most deplorable from want of food, and little or no medical advice or medicine. Several of the English residents died : among these was Dr. Kenny. The fever was believed to be the genuine black-vomit fever ; it proved contagious, almost without exception, to the nurses of the sick."

Such is the substance of Mr. Rendall's letter.

Two days subsequently, Mr. Macaulay, Queen's Commissary Judge at the Cape Verde Islands, wrote to Lord Aberdeen upon the same subject, going over the same ground as Mr. Rendall, and confirming, in most particulars, his statements. Both gentlemen imply, and strongly express their regret, that the medical officers of the Eclair were not so frank and communicative, in their report of the character of the fever on board, as they ought to have been. For example, no mention was made by them of black vomit having been observed in any case, and they uniformly declared the disease to be only the ordinary remittent fever of the coast of Africa. Mr. M. concludes his letter in these words :

"It is a satisfaction to know that the 'Eclair' had left Bona Vista nearly a month before any case of 'Eclair' fever exhibited itself in the town.* This circumstance, coupled with the fact that no injury whatever had resulted from the unrestricted intercourse which had subsisted during the whole of the 'Eclair's' stay in the harbour, between the officers and men (not in hospital at the fort) and their friends on shore, proves, I think clearly, that had the building which was so long used as a fever hospital been properly fumigated and purified prior to its re-occupation ; and had the two soldiers, who were there seized with the fever, been kept (like the fever patients of the Eclair) from all intercourse with the densely-peopled and closely-built town, no bad consequences would have been experienced at Bona Vista from the visit of the 'Eclair,' and the kindness and hospitality which were there extended to her."

There has been another letter received from Mr. Rendall, dated 9th

---

* This statement is, it will be observed, at variance with what Mr. Rendall says.

March. By it we learn that "the sickness still continues in the villages of the interior of the island (Bona Vista), and that the number of deaths has amounted to upwards of 400."

Here we must not omit to mention that Dr. Stewart states in his Report (on the authority, we have understood, of the Consul's son, then recently arrived at Bona Vista) that the inhabitants of that town were healthy during the stay of the Eclair, but that yellow fever existed at the time in the adjoining island of Porto Praya. If this was really and truly the case, we might fairly conclude that an unhealthy condition of the atmosphere, such as must always powerfully favour and promote the diffusion of febrific miasms, prevailed over the Cape de Verde Islands at the very time when the fever seems to have been imported by the infected steamer into Bona Vista. We say, "*seems* to have been imported"—not that we have almost any doubt in our own mind that such was actually the case; but merely because we are unwilling to commit ourselves to any decided opinion upon the point in question, until we have the opportunity of perusing a medical report upon the state of things at that island subsequent to the departure of the Eclair. This, we are glad to learn, will ere long be the case; as Dr. Mac William, well known as the author of an excellent work on the recent Niger expedition, and who has had an extensive acquaintance with fever on the coast of Africa, has been sent out by the Admitalty to Bona Vista to investigate fully everything connected with the destructive pestilence with which it has been visited. Meanwhile, we would suggest to the surgeon of the "Growler" how much it might serve to elucidate the entire history of the "Eclair" fever, if he were to communicate to the public a report of the health of the former vessel for some time before her departure from Sierra Leone until the time when she was paid off at Woolwich. At the same time, we may state that it is our intention to bring the subject of Quarantine under the notice of our readers in our next number, having had the recent very able Report of the French Academy upon that question for some months past under consideration.

It would not be fair to the memory of a most meritorious officer and excellent man, if we closed this article without expressing the gratification we have derived from the perusal of the "Notes and Recollections" of the late Dr. Fergusson. With the prevailing spirit of its contents we cordially and completely agree.

